# Comparison of ankle-brachial pressure index and pulse wave velocity as markers of cognitive function in a community-dwelling population

**DOI:** 10.1186/1471-244X-10-46

**Published:** 2010-06-10

**Authors:** Norio Sugawara, Norio Yasui-Furukori, Takashi Umeda, Ayako Kaneda, Yasushi Sato, Ippei Takahashi, Masashi Matsuzaka, Kazuma Danjo, Shigeyuki Nakaji, Sunao Kaneko

**Affiliations:** 1 Department of Psychiatry, Hirosaki-Aiseikai Hospital, Hirosaki, Japan; 2 Department of Neuropsychiatry, Hirosaki University School of Medicine, Hirosaki, Japan; 3 Department of Social Medicine, Hirosaki University School of Medicine, Hirosaki, Japan

## Abstract

**Background:**

Vascular factors have been implicated in the development of cognitive decline and dementia. The purpose of this study is to determine the association of the Ankle Brachial pressure Index (ABI) and brachial-ankle Pulse Wave Velocity (ba-PWV) to cognitive impairment in a community-dwelling population.

**Methods:**

The ABI and ba-PWV were measured using the volume-plethymographic apparatus in 388 subjects aged 60 years old and over. The Mini-Mental State Examination was also employed to measure global cognitive status. The effectiveness of the ABI and ba-PWV as putative markers of cognitive impairment were determined by using a multiple logistic regression analysis after adjusting for confounding factors.

**Results:**

Subjects with poor cognition were significantly older and less well educated than those with normal cognition. According to the multiple logistic regression analysis, the lowest ABI tertile was found to be a significant independent risk factor (OR = 3.19, 95% CI = 1.30 to 7.82) of the cognitive impairment, whereas the highest brachial-ankle PWV tertile was not.

**Conclusions:**

A low ABI was an independent risk factor for cognitive impairment in community-dwelling older populations, whereas a high ba-PWV may not be. Further research will be required to analyze ABI and PWV with greater accuracy.

## Background

The cognitive impairment is an important health issue contributing to disability [1], morbidity [2] and mortality [3]. The identification of clinical markers predicting cognitive impairment in elderly is often considered useful in easing the public health burden of poor cognition, as aging is associated with an increased risk of cognitive impairment.

Vascular factors have been implicated in the development of cognitive decline [4] and dementia [5]. As common quantitative assessments of arterial health, the Ankle Brachial pressure Index (ABI) and Pulse Wave Velocity (PWV) are often considered to determine blocked arteries and arterial stiffness respectively, using non-invasive measures.

The ABI is the ratio of the ankle and the brachial systolic blood pressure and is used to assess the severity of arterial occlusion in the leg, and a reduction of ABI suggests the presence of peripheral arterial disease due to atherosclerosis. It is also known that atherosclerosis in the lower legs represent just one manifestation of a similar pathology in other arterial systems [6,7]. A number of previous studies [4,8,9] on the relationship between ABI and cognitive function were carried out, in population-based designs. Breteler et al [10] had observed that peripheral arterial disease was associated with lower average mental status scores and shifts in score distribution. In a large US community-based study [8], a low ABI was associated with the decline of cognitive function over 7 years of follow up. Other cohort studies including the Edinburgh Artery study and the Honolulu-Asia Aging study, showed that a lower ABI had predictive value for future risk of cognitive impairment [4] and increased the risk of dementia [9].

Another assessment for arterial stiffness is PWV, in which the velocity of the pulse wave is measured as it travels a given distance between 2 sites along the arterial system. Since Mizushima et al. [11] first reported that PWV was higher for individuals with vascular dementia than those without dementia; the PWV has been recognized as a risk factor of dementia [12,13]. According to a result obtained in a cross-sectional design study [14-16], a higher PWV has also been related to a decrease in performance on screening measures of cognitive function, such as the Mini-Mental State Examination (MMSE). The longitudinal effects of PWV to verbal learning, delayed recall, non verbal learning [17] and global cognitive function [18] were also reported.

These findings contribute to the growing body of evidence on correlation between arterial stiffness and cognitive impairment. Recently, a measurement device which can simultaneously measure the ABI and brachial-ankle PWV has become available [19]. However, the association between these 2 putative markers for the assessment of cognitive impairment in a community-dwelling population has not yet been determined.

In this study, the ABI and ba-PWV were compared as markers of cognitive impairment in a community-dwelling population, and MMSE was used to assess cognitive status. The ABI and PWV were measured as indicators of arterial structure and function. The present study is considered as the report to obtain a comparative data between the ABI and ba-PWV as markers of cognitive function in a community-dwelling population.

## Method

### Participants

The subjects were 388 volunteers (60 years old and over; 139 males and 249 females) who participated in the Iwaki Health Promotion Project in 2008. The data collection for this study was approved by the Ethics Committee of Hirosaki University School of Medicine and all subjects had provided written informed consent before participating in the project. The demographic data (age, sex, amount of education) and life style (smoking, drinking) were obtained from self-questionnaires and interviews. The height and weight of subjects were measured, and body mass index (BMI) was calculated. Low-density lipoprotein (LDL) cholesterol, triglyceride and HbA1c were also measured by standard analytical techniques.

### ABI and brachial-ankle PWV measurements

The ankle and brachial pressures were measured using the volume-plethymographic apparatus (form PWV/ABI, COLIN Co. Ltd., Tokyo, Japan). In addition to recording the limb lead ECG and mechano-cardiograms were simultaneously recorded by attaching blood pressure cuffs with a tonometic sensor to the upper arm and ankle.

The ABI was determined as the ratio of ankle systolic blood pressure to brachial systolic blood pressure. To calculate the ABI, the brachial pressure was measured in the left arm, and the ankle pressure was measured for both the left and right sides with subjects in the supine position and the lowest value of the ABI was taken [9].

The brachial-ankle PWV (ba-PWV) was calculated by time-phase analysis. The time interval between the wave front of the brachial waveform and that of the ankle waveform was defined as the time interval between the brachium and ankle (ΔTba). The distance between sampling points of ba-PWV was calculated automatically according to the height of the subject. The path length from the suprasternal notch to the brachium (Lb) was obtained from superficial measurements and was expressed using the following equation: Lb = 0.2195 × height of the patient (in cm) -2.0734. The path length from the suprasternal notch to the ankle (La) was obtained from superficial measurements and was expressed using the following equation: La = (0.8129 × height of the patient (in cm)+12.328). Finally, the following equation was used to obtain ba-PWV: ba-PWV = (La-Lb)/ΔTba [20]. In this study, a higher value of ba-PWV was employed.

### Assessment of cognitive function

The Mini-Mental State Examination (MMSE) was given to all participants to measure their global cognitive status. This test assesses orientation to place and time, short term memory, episodic long-term memory, subtraction, ability to construct a sentence, and oral language ability. The maximum score was set as 30, and poor cognition was defined as a score of less than 24 [21]. Subjects were divided into 2 groups: control group (MMSE≧24) and the group with subjects having a cognitive impairment (MMSE≦23).

### Statistical Analysis

Data are presented as mean ± SD. A value of p < 0.05 was considered significant. Student's unpaired t test for continuous variables or chi-square test for categorical variables was used to evaluate the difference in variables between subjects with MMSE≦23 and those with MMSE≧24. The subjects were divided into 3 groups according to their ABI and ba-PWV tertiles, and a multivariate logistic regression analysis was applied to assess the usefulness of the ABI and ba-PWV measurements as markers of cognitive impairment both crude and adjusted conditions for confounding factors (age, sex, amount of education, smoking status, habitual alcohol intake, body mass index, LDL-cholesterol, tryglyceride, HbA1c, systolic blood pressure, pulse pressure) [22]. The data were analyzed using the SPSS-PC-software for Windows, Version 12.0.

## Results

### Sample characteristics

The subjects were divided into 2 groups according to their MMSE scores (poor cognition, MMSE≦23, n = 41; control, MMSE≧24, n = 347). The clinical characteristics of subjects are listed in Table 1. Subjects with poor cognition were significantly older and less educated than the control group. No other differences on all the other characteristics including the ba-PWV were observed.

**Table 1:** Demographic Characteristics of the Subjects

	poor cognition (n = 41)	control (n = 347)
MMSE*	21.5 ± 1.8	28.0 ± 2.0
Age (years old) *	70.1 ± 4.9	68.3 ± 5.6
Sex	male 21, female 20	male 118, female 229
Amount of education (years) *	9.3 ± 1.9	10.4 ± 2.1
Current smoking (0; no, 1; yes)	Yes 3, No 38	Yes 30, No 317
Habitual alcohol intake (0; no, 1; yes)	Yes 22, No 19	Yes 131, No 216
Body mass index (kg/m^2^)	23.5 ± 3.3	23.3 ± 2.9
LDL-cholesterol (mmol/L)	116.1 ± 26.3	118.9 ± 24.4
Triglyceride (mmol/L)	92.4 ± 47.4	92.6 ± 50.9
HbA1c (%)	5.4 ± 0.8	5.3 ± 0.6
Systolic blood pressure (mmHg)	137.6 ± 17.8	136.8 ± 17.3
Pulse pressure (mmHg)	57.0 ± 12.1	57.2 ± 12.0
Ankle-Brachial pressure index	1.09 ± 0.08	1.11 ± 0.09
Pulse Wave Velocity (mm/s)	1839 ± 413	1737 ± 298

Values are mean ± S.D. Student's unpaired t test for continuous variables or chi-square test for categorical variables was used to evaluate the differences between poor cognition (MMSE≦23) and control (MMSE≧24). *: p < 0.05

### Effect of ABI, PWV and education on cognitive decline

The subjects were grouped according to their ABI tertile (first quartile 0.66 to 1.08, n = 129; second tertile 1.08 to 1.15, n = 130; third tertile 1.15 to 1.59, n = 129) and according to their ba-PWV tertile (first tertile 1159 to 1615 cm/s, n = 129; second tertile 1616 to 1895 cm/s, n = 130; third tertile 1899 to 3352 cm/s, n = 129). Table 2 presents the results of the multivariate logistic regression analysis used to assess the usefulness of the ABI and the brachial-ankle PWV as markers of the cognitive impairment. After adjusting for confounding factors, the 1^st ^quartile of ABI (OR = 3.19, 95% CI = 1.30 to 7.82) was shown to be an independent risk factors. In crude odds ratio, the 1^st ^quartile of ABI (OR = 2.18, 95% CI = 0.98 to 4.87) approached statistical significance. Under both crude and adjusted conditions, amount of education consistently showed lower risk of cognitive impairment. There were no other significant risk factors of cognitive decline.

**Table 2:** Risk factors associated with having lower MMSE (< 24) estimated by logistic regression analysis.

Parameter		Crude odds ratio	p Value	Adjusted odds ratio	p Value
**Amount of education (years)**		0.78 (0.66-0.91)	< 0.01	0.78 (0.65-0.93)	< 0.01
**Ankle-Brachial pressure index**					
1st	Tertile	2.18 (0.98-4.87)	< 0.10	3.19 (1.30-7.82)	< 0.05
2nd	Tertile	1.10 (0.45-2.69)	N.S.	1.37 (0.54-3.48)	N.S.
3rd	Tertile	1.00		1.00	
**Pulse Wave Velocity**					
1st	Tertile	1.00		1.00	
2nd	Tertile	1.57 (0.68-3.63)	N.S.	1.43 (0.48-4.23)	N.S.
3rd	Tertile	1.67 (0.73-3.83)	N.S.	1.50 (0.58-3.85)	N.S.

Mean (95% confidence interval)

## Discussion

The ABI and PWV are the markers of atherosclerosis and arterial stiffness respectively. In the present research, their associations with cognitive impairment were examined through cross-sectional assessment among community-dwelling population. The cognitive decline was found to be 3.19 times more prevalent among subjects in the 1st tertile of ABI (0.66 to 1.08). On the other hand, no PWV tertiles showed an association with cognitive impairment in either crude or adjusted conditions. This research is considered as the report suggesting a usefulness of ABI and PWV as markers of cognitive function in a community-dwelling population.

The measurement of the ABI provides one of the most practical means to objectively assess the presence of atherosclerosis. A number of previous studies [4,8,9] suggested that, even at subclinical levels, atherosclerosis is associated with an increased risk of progressive cognitive decline and ABI might be of clinical value in identifying older people who are at increased risk of cognitive impairment. The mechanism leading to the association between ABI and cognitive function still remains unclear. However, because ABI is a marker of structural and functional changes in the arterial vessels, several mechanisms may explain an association between atherosclerosis and dementia.

One explanation is that sub-clinical cerebrovascular disease, such as silent cerebral infarctions may mediate the association between atherosclerosis and cognitive impairment. In the present research, brain imaging was not carried out on subjects, thus this was not evaluated. Also atherosclerosis may induce cerebral hypo-perfusion leading to cerebral hypoxia. These conditions may destabilize neurons and synapses and evolve in a neurodegenerative process characterized by formation of senile plaques, neurofibrillary tangles, and amyloid angiography [5,23,24]. Another explanation is that atherosclerosis and cognitive decline may share common genetic or environmental risk factors such as smoking, hypertension and hyper-lipidemia in the causal pathway [25]. The ABI measured in this study may reflect behaviour in early life that in turn influenced cognitive function. Although adjustment for cardiovascular risk factors in our study did not change the association between atherosclerosis and cognitive impairment as expected, this explanation still cannot be excluded.

In this study, we did not find an association between PWV and risk of cognitive impairment. Some findings obtained from other studies were found to support our results. For example, Dhoat et al [26] did not find a difference of PWV between dementia and control groups. In a large study of older Dutch population [27], arterial stiffness was not identified as an independent risk factor of cognitive decline neither. However, a number of studies [14-16] showed inverse relationships between PWV and MMSE. The brachial-ankle PWV (ba-PWV), which reflects left ventricular structure and function, was used in the present research to assess the arterial stiffness, whereas the carotid-femoral PWV (cf-PWV), a marker of arterial stiffness over the central elasticity was used in various studies. Ba-PWV is affected by not only central elastic arteries but also peripheral muscular ones [28]. Although ba-PWV correlates well with cf-PWV with a correlation coefficient around 0.75-0.87 [20,29] and cf-PWV is the main determinant of ba-PWV [29], there is a substantial difference between the two techniques over the range of measurement [30].

In order to assess cognitive status, the MMSE was employed in this study. The MMSE score does not always reflect the cognitive function exactly, as it is sometimes known to be influenced by the education level of the subject. In the present study, the subjects with a normal MMSE score were significantly more educated than those with impaired MMSE scores, and the amount of education was found to have significant association with the MMSE score. This finding was consistent with that of previous reports. In Baltimore longitudinal Study of aging [17], persons with higher baseline PWV also exhibited prospective decline on tests of verbal learning and delayed recall, nonverbal memory, and the Blessed I-M-C Test. However, the same test did not show the association between PWV and MMSE.

As all subjects were volunteers who had interests in their health, they may be healthier than those who were not involved in this study. Thus, those who were not would be more likely to do poorly on a community-based cognitive assessment [31]. A "selective bias" must also be considered in studies of older persons. Those with severe arterial stiffness might not live to old age. However, the current findings indicate that a low ABI is an independent risk factor for cognitive impairment.

In the present study, the brain imaging was not carried out, thus Alzheimer's disease (AD) was not excluded completely. The aetiology of AD is different from vascular-type dementia. Including subjects with AD could obscure the association between vascular factors and cognitive function. Nevertheless, the result of this study, which showed cognitive impairment in subjects with lower ABI, suggests the predictive value of ABI.

## Conclusions

In this study, we determined the association between ABI and PWV to cognitive impairment in cross-sectional study. A score in the lowest ABI tertile is an independent risk factor for cognitive impairment, whereas a score in a high ba-PWV tertile was not. However, we are not able to completely rule out an association between increased PWV and risk of cognitive impairment due to a number of factors including the measurement site of PWV, use of the MMSE, having a relatively small sample size and the possibility of a selection bias. Furthermore, a comparison between ABI and PWV was not performed in the longitudinal situation. There is still no cure for dementia, and the risk factors of cognitive decline must be identified to delay its progression. Therefore, further investigation is needed to obtain clear associations between them in the future.

## Competing interests

The authors declare that they have no competing interests.

## Authors' contributions

NS conceived the study, designed the study, conducted the statistical analysis, interpreted the data and wrote the initial draft of the manuscript. SK had full access to all of the data in the study and takes responsibility for the integrity of the data and the accuracy of the data analysis. SK, SN and NYF contributed to study design and assisted in drafting the manuscript. TU and IT completed initial survey construction, recruitment of participants. AK, YS, KD and MM participated in the data collection, and the interpretation of the results. All authors have approved the manuscript.

## Pre-publication history

The pre-publication history for this paper can be accessed here:

http://www.biomedcentral.com/1471-244X/10/46/prepub
